# Uncoupling of Satellite DNA and Centromeric Function in the Genus *Equus*

**DOI:** 10.1371/journal.pgen.1000845

**Published:** 2010-02-12

**Authors:** Francesca M. Piras, Solomon G. Nergadze, Elisa Magnani, Livia Bertoni, Carmen Attolini, Lela Khoriauli, Elena Raimondi, Elena Giulotto

**Affiliations:** Dipartimento di Genetica e Microbiologia “Adriano Buzzati-Traverso”, Università di Pavia, Pavia, Italy; Murdoch Children's Research Institute, Australia

## Abstract

In a previous study, we showed that centromere repositioning, that is the shift along the chromosome of the centromeric function without DNA sequence rearrangement, has occurred frequently during the evolution of the genus *Equus*. In this work, the analysis of the chromosomal distribution of satellite tandem repeats in *Equus caballus, E. asinus, E. grevyi*, and *E. burchelli* highlighted two atypical features: 1) several centromeres, including the previously described evolutionary new centromeres (ENCs), seem to be devoid of satellite DNA, and 2) satellite repeats are often present at non-centromeric termini, probably corresponding to relics of ancestral now inactive centromeres. Immuno-FISH experiments using satellite DNA and antibodies against the kinetochore protein CENP-A demonstrated that satellite-less primary constrictions are actually endowed with centromeric function. The phylogenetic reconstruction of centromere repositioning events demonstrates that the acquisition of satellite DNA occurs after the formation of the centromere during evolution and that centromeres can function over millions of years and many generations without detectable satellite DNA. The rapidly evolving *Equus* species gave us the opportunity to identify different intermediate steps along the full maturation of ENCs.

## Author Summary

Centromeres are the functional elements controlling chromosome segregation during cell division. Vertebrate centromeres, which typically contain large amounts of tandem repeats (satellite DNA), are highly conserved for function but not for DNA sequence, suggesting that centromeric function is mainly determined by epigenetic factors. Evolutionary centromere repositioning is the shift of a centromere to a new position in the absence of structural chromosome rearrangements. In previous work, we demonstrated that centromere repositioning was exceptionally frequent during the evolution of the genus *Equus* (horses, asses, and zebras). In the present paper, we show that several *Equus* centromeres, including all the previously described evolutionary new centromeres, are apparently satellite-free, supporting the idea that large blocks of repeats are not necessarily required for the stability of centromeres. Our results suggest that centromere repositioning might be a two-step event: first, a neocentromere arises in a satellite-less region; satellite repeats may then colonize this repositioned centromere at a later stage, giving rise to a “mature” centromere. The rapidly evolving *Equus* species gave us the opportunity to catch snapshots of several evolutionary novel centromeres in different stages during their maturation.

## Introduction

Centromeres, cytologically appearing as visible primary constrictions in metaphase chromosomes, are essential for the proper segregation of sister chromatids during cell division. They are the sites of kinetochore assembly and spindle fiber attachment and consist of protein-DNA complexes, in which the DNA component is typically characterized by the presence of extended arrays of tandem repeats (called satellite DNA). Satellite DNA, initially purified by density gradient centrifugation experiments [1],[2], is organized as long arrays of head-to-tail repeats, located in the constitutive heterochromatin.

Two observations have suggested that, although satellite DNA sequences and centromeres are often associated with one another, satellite DNA itself is not required for centromere function. Firstly it became clear that, in spite of the proposed involvement of these sequences in a highly conserved cell division-related function, they are remarkably different among different species. This observation, known as the “centromere paradox”, pointed to epigenetic factors as being responsible for centromere function through binding of the DNA with kinetochore proteins [3]. Secondly, and perhaps more influentially, the group of Choo [4] and subsequently several other groups [5] were able to identify and analyse neocentromeres in rare human clinical material. The analysis of neocentromeres demonstrated that full centromere function can occur in the absence of the sequence organization characteristic of most natural centromeres and that a DNA fragment may acquire centromere function without any sequence alteration, a phenomenon defined “centromerization” [6]. The existence of neocentromeres and the rapid evolution of centromeric DNA suggested that an epigenetic mark rather than DNA sequence determines centromere function. The identity of this mark remains a matter of investigation. Some have argued that the mark is the ability to be bound by CENP-A, a centromere specific variant of the histone H3 [3], while others have argued that the mark is a feedback loop in which centromere stretching at metaphase plays a critical role [7].

Another phenomenon supporting the epigenetic nature of centromeres is evolutionary repositioning, that is the shift along the chromosome of the primary constriction together with the centromeric function. Comparative studies of chromosomes in primates, other placental mammals, marsupials and birds have demonstrated that the positioning of centromeres can change over the course of evolution, in the absence of any other significant and detectable change in marker order along the chromosome, generating evolutionary new centromeres (ENCs) [8]–[13]. It has been proposed that the initial event of evolutionary repositioning may be the loss of function of the original centromere followed by the gain of epigenetic signals in a non-centromeric position. Such a sequence of events would lead to the formation of a centromere in a new chromosome region devoid of satellite DNA [10],[11],[14]. This “young” neocentromere may then gradually accumulate, during several successive generations, repetitive DNA through various recombination-based mechanisms. These events would lead to the formation of a centromere in a new permissive chromosome region devoid of satellite DNA, without involvement of DNA sequence alterations. Since all natural centromeres described so far, including ENCs, contain satellite DNA sequences, Marshall and co-workers [5] proposed that satellite sequences are incorporated at repositioned centromere sites, because they probably confer an adaptive advantage possibly by increasing the accuracy of chromosome segregation. Alternatively, the accumulation of satellite sequences may simply be a neutral process driven by the presence of heterochromatin in the centromeric DNA.

In this scenario, we might expect to find evolutionarily immature centromeres, lacking satellite DNA, in rapidly evolving species. Equids are a representative example of quickly radiating organisms; the eight living species of the Equidae family belong to the genus *Equus* and comprise: two horses (*E. caballus* and *E. przewalskii*), two Asiatic asses (*E. kiang* and *E. hemionus*), one African ass (*E. asinus*) and three zebras (*E. grevyi, E. burchelli* and *E. zebra*). The *Equus* species shared a common ancestor about 2–3 million years ago and the extant species emerged about 1 million years ago, that is in a very short evolutionary time [15]. These animals are valuable for comparative cytogenetics because, in spite of their recent divergence, morphological similarity and capacity to interbreed, their karyotypes differ extensively [16]–[18]. The variation involves both the structure and the number of chromosomes, which ranges from 32 in *E. zebra* to 66 in *E. przewalskii*. Cross-species chromosome painting has confirmed the great karyotypic variability of this genus [19]. In addition, we have shown that at least nine centromere repositioning events took place during the evolution of this genus, six of which occurred in *E. asinus* (donkey) [12],[20] and one of which occurred in horse chromosome 11 (ECA 11). These results demonstrate that the phenomenon of centromere repositioning played a key role in the rapid karyotypic evolution of the equids and point to these species as an ideal model system for the analysis of neocentromere formation and centromere evolution. The observation that a number of evolutionary novel centromeres are present in the rapidly evolving *Equus* species, prompted us to investigate their sequence organization in order to ascertain whether any of them lack satellite DNA, in agreement with the above described model of centromere shift during evolution.

The first part of this analysis was the determination of the DNA sequence of the evolutionary new centromere on horse chromosome 11, which demonstrated that this centromere lacks any satellite DNA sequences [21]. This observation strongly supports the hypothesis that this centromere was formed recently during the evolution of the horse lineage and, in spite of being functional and stable in all horses, did not acquire all the marks typical of mammalian centromeres, probably representing the first example of an evolutionary “immature” centromere. Here, with the goal of identifying other possible cases of satellite-less ENCs, we performed an extensive cytogenetic analysis of the organization of centromeric sequences in four *Equus* species: the domestic horse (*E. caballus*), the domestic donkey (*E. asinus*), and two zebras (*E. grevyi* and *E. burchelli*). The results suggest that several such “immature” ENCs may indeed be present in these species. The presence of so many apparently-satellite-free evolutionary new centromeres suggests that, at least in this genus, there is no adaptive requirement for the acquisition of centromeric satellite DNA once neocentromeres are formed.

## Results

### Localization of the Two Major *Equus* Satellite DNA Sequence Families

Two satellite DNA sequences were previously isolated from a horse genomic library in lambda phage [22] using two procedures. A satellite (37cen) was identified in a phage clone containing a large restriction fragment following double digestions with frequently cutting restriction enzymes. The second satellite (2PI) was isolated as a by-product of a screen of the same library for minisatellites. The phage clones were sub-cloned in plasmid vector and sequenced. The 37cen sequence, consisting of a 221 bp repeat (Accession number: AY029358), is 93% identical to the horse major satellite family independently identified by Wijers and colleagues [23] and by Sakagami and co-workers [24]. The 2PI sequence, consisting of a 23 bp repeat (Accession numbers: AY029359S1 and AY029359S2), belongs to the e4/1 family described by Broad and colleagues [25],[26] and shares 83% identity with it. Zoo-blot analysis showed that the two horse satellites are undetectable in cow, goat, sheep, man, dog, mouse, Syrian hamster, mediterranean fruit fly and yeast, while they are present in several species of the genus *Equus,* including *E. caballus*, *E. asinus*, *E. grevyi* and *E. burchelli* (data not shown).

**Figure 1:**
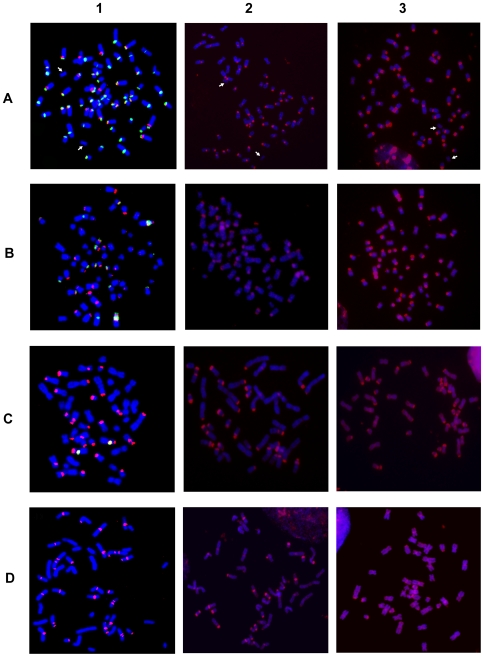
Hybridization of satellite DNA probes with chromosomes from four *Equus* species. Column 1: two color FISH on horse (A), donkey (B) Grevy's (C) and Burchelli's (D) zebras chromosomes. The FISH probes were the 37cen (green) and the 2PI (red) satellite DNA sequences; co-hybridization of both probes results in yellow signals. Column 2: horse (A), donkey (B), Grevy's (C), and Burchelli's (D) zebra metaphases hybridized in high stringency conditions with autologous genomic DNA, which identifies regions containing very abundant tandem repeats. Column 3: the same hybridization of column 2 performed in low stringency conditions. The white arrows in (A) point to chromosomes 11, which is the only horse chromosome pair lacking any hybridization signal.

**Figure 2:**
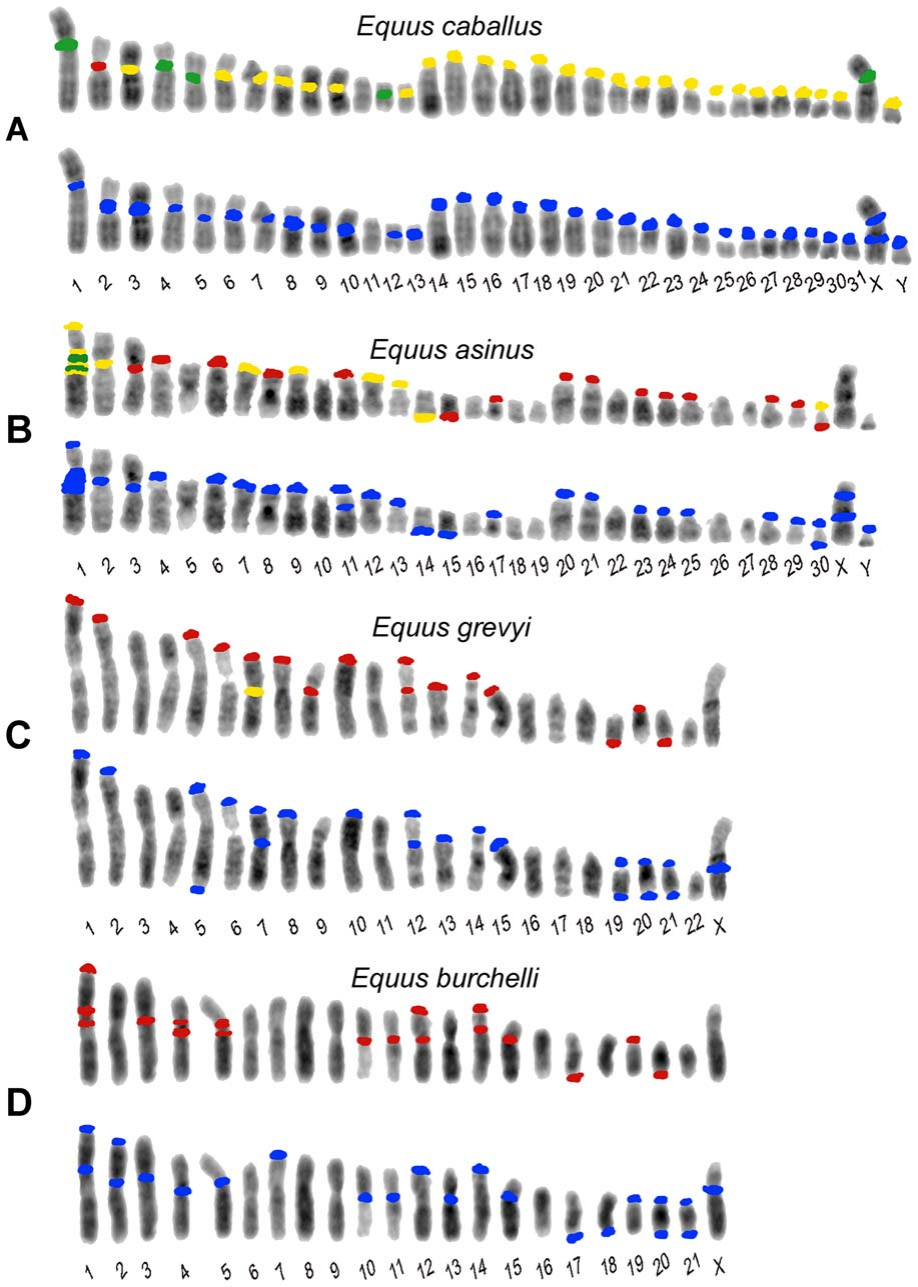
Schematic representation of the FISH signals. Distribution of FISH signals on horse (A), donkey (B), Grevy's (C), and Burchelli's (D) zebras chromosomes. At least 20 metaphases from each hybridization experiment were analyzed, examples of which are shown in Figure 1. Hybridization positive loci have been marked in different colors on banded karyotypes from each species: loci hybridizing with the 37cen probe only are labelled in green, 2PI positive loci are labelled in red and loci hybridizing with both 37cen and 2PI are labelled in yellow. Hybridization with genomic DNA probes, detecting total satellite DNA, is marked in blue. The differences in signal intensity among the sites, visible in Figure 1, are not reported here.

To localize these satellites, two color FISH experiments were performed using the 37cen and 2PI sequences as probes on metaphase chromosomes from *E. caballus* (ECA, horse) (Figure 1A, column 1), *E. asinus* (EAS, domestic donkey) (Figure 1B, column 1), *E. grevyi* (EGR, Grevy's zebra) (Figure 1C, column 1) and *E. burchelli* (EBU, Burchelli's zebra) (Figure 1D, column 1). The chromosomal distribution of the two satellites was analyzed from single and double color FISH experiments and the results are schematically reported in the top rows of each panel of Figure 2A–2D.

In *E. caballus* (Figure 2A, top row), the majority of centromeres contained both satellites (yellow), five chromosomes (1, 4, 5, 12 and X) showed only 37cen signals (green) and chromosome 2 showed only the 2PI signal (red). The centromere of chromosome 11 was the only one lacking any signal. Thus, 37cen was localized at the centromeric region of all chromosomes except 2 and 11; these results are essentially in agreement with those from Sakagami and colleagues [24] who localized a satellite DNA sequence, belonging to the same family, on all horse centromeres except three; this discrepancy is not surprising, considering that we used fluorescence-based approaches whereas they used radioactive probes, which are known to be less sensitive. The 2PI sequence was present at the centromere of all the acrocentric horse chromosomes, as well as at the centromere of eight meta- or submeta-centric chromosomes (2, 3, 6, 7, 8, 9, 10 and 13). Thus, all centromeres have either one or both satellites while ECA11 is the only *E. caballus* chromosome lacking signals from both satellites.

In *E .asinus* (Figure 2B, top row), the distribution of the two satellites was different when compared to *E. caballus*; in fact, several chromosomes, while lacking satellite signals at their centromeres, contained such signals at one non-centromeric terminus. In particular, the 37cen sequence was localized on one telomeric end of six meta- or submeta-centric chromosome pairs (1p, 7p, 9p, 12p, 13p, and 14q) and in the centromeric region of three chromosomes only (1, 2 and 30), chromosome 1 showing a very large subcentromeric signal; thus, in chromosome 1, this probe recognized both the p arm terminus and the extended subcentromeric heterochromatic region. The 2PI satellite was located at one terminus of thirteen meta- or submeta-centric donkey chromosomes (1p, 4p, 6p, 7p, 8p, 9p, 11p, 12p, 13p, 17p, 14q, 15q and 30q) and on the centromeric region of eleven chromosomes (1, 2, 3, 20, 21, 23, 24, 25, 28, 29 and 30), the extended chromosome 1 subcentromeric region showing two clearly distinguishable separate signals.

In *E. grevyi* (Figure 2C, top row), 37cen was much less represented, being detectable only on the centromeric region of the submetacentric chromosome 7. Conversely, 2PI was abundant, since it was found in one non-centromeric end of thirteen chromosomes (1p, 2p, 5p, 6p, 7p, 8p, 10p, 12p, 13p, 14p, 15p, 19q and 21q) and on the centromeric region of chromosomes 7, 9, 12 and 20; thus, chromosomes 7 and 12 contain 2PI sequences both at the centromere and at the p arm terminus.

Finally in *E. burchelli* (Figure 2D, top row), the 37cen sequence was undetectable whereas the 2PI sequence was abundant, hybridization signals being present on the centromere of ten chromosomes (1, 3, 4, 5, 10, 11, 12, 14, 15 and 19); on chromosomes 1 and 5, an additional signal was clearly detectable in the subcentromeric region and on chromosome 4 in the proximal region of the short arm. On EBU 1p, 12p, 14p, 17q and 20q, terminal 2PI signals were also present; therefore, chromosomes 1, 12 and 14 contain this satellite both at the centromere and at one end.

A further point to be remarked is that fourteen EGR and EBU meta- or submeta-centric autosomes are syntenic, as shown by chromosome painting [27], share the same banding pattern, being presumably derived from fusion of ancestral acrocentrics. However, we observed that the majority of these chromosomes showed a different distribution of the 2PI satellite; these EGR/EBU chromosomes were: 1/1, 2/2, 3/3, 5/5, 6/6, 7/7, 8/8, 10/10, 12/13, 14/15, 15/16, 19/19 and 20/20. Only the syntenic chromosomes EGR 16 and EBU 18 have the same satellite distribution. The discrepancy in the distribution of satellite DNA sequence that we observed may be mainly ascribed to a differential retention of repetitive sequences at sites corresponding to centromeres of ancestral acrocentric chromosomes.

The data reported in Figure 1, column 1, were obtained by high stringency hybridization (see Materials and Methods). Hybridizations at low stringency were also performed and the results were super-imposable to those obtained at high stringency except for a higher background (data not shown).

### Localization of Other Satellite Sequences

The absence of detectable 37cen and 2PI FISH signals from the centromeres of *E. caballus* (horse) chromosome 11 and of several *E. asinus* (donkey), *E. grevyi* (Grevyi's zebra) and *E. burchelli* (Burchelli's zebra) chromosomes, raises the question whether satellite DNA, belonging to other families, might be present at such centromeres. To investigate this possibility, we performed FISH analysis on the chromosomes of the four species, using their total genomic DNA as probe, at both high and low stringency (Figure 1, columns 2 and 3 and bottom rows of Figure 2A–2D). Also in this case, the data obtained with high and low stringency were essentially super-imposable, except for a higher background in the latter (compare columns 2 and 3 in Figure 1). This procedure can allow the identification of regions containing very abundant tandem repeats due to the different hybridization kinetics of highly reiterated sequences versus single copy DNA. This approach is especially effective for the identification of satellite DNA in the *Equus* species, providing a resolution comparable to that of FISH performed with cloned satellite probes, as clearly shown by the high specificity of the pattern of hybridization signals and by the overall similarity of signal distribution in the top and bottom rows of each panel in Figure 2. The particular adequacy of this approach to localize satellite sequences on *Equus* chromosomes may be due to a high degree of homogeneity in the organization of tandem repeat arrays in these genomes.

In the horse, when the chromosomes were hybridized with total horse genomic DNA (Figure 1A, column 2 and column 3), all the centromeres, except the one of chromosome 11 (white arrows), were labelled with specific signals; the distribution of these signals (Figure 2A, bottom row) corresponded to that observed with a 1∶1 mix of the single satellite probes (Figure 2A, top row), with one exception consisting in a faint interstitial signal on the long arm of the X chromosome detectable only by hybridization with genomic DNA. This observation indicated that satellite sequences other than 37cen or 2PI are present on chromosome X. Strikingly, we obtained a similar pattern of hybridization when we used donkey, Grevy's zebra or Burchelli's zebra genomic DNAs as probes on horse chromosomes; however, a certain degree of variation in signal intensities was observed on specific sites (data not shown). Very similar hybridization patterns were also observed on donkey and zebra chromosomes probed with their own genomic DNA or with genomic DNA from the other species (data not shown). These results indicated that 37cen and 2PI are the most abundant satellite sequences in these four species.

Also in the donkey (Figure 2B), Grevy's zebra (Figure 2C) and Burchelli's zebra (Figure 2D) the distribution of the FISH signals using the two approaches was not exactly comparable. In fact, following hybridization with genomic DNA, a few sites of hybridization were observed that were not detected with the 37cen and 2PI probes; these (Figure 2B–2D) involved chromosomes X in all the three species, Y in the donkey (no information on the Y chromosome of EBU and EGR is available), EAS 11cen, EGR 5qtel, EGR 19cen, EGR 20qtel, EGR 21cen, EBU 2cen, EBU 2ptel, EBU 7ptel, EBU 13cen, EBU 18qtel, EBU 20cen, EBU 21cen, EBU 21qtel. It must be mentioned here that the telomeric signal on EGR 5q represents a polymorphic marker since it was repetitively observed on one only of the two homologues. In addition, EGR 9, EBU 12 and EBU 14 showed hybridization signals with the cloned satellite probes and not with genomic DNA; this might have been due to a relatively low abundance of the repeats located at these sites. This observation indicates that we cannot rule out the presence of low abundance tandem repeats at some of the centromeres where FISH signals were not detected. Altogether these results suggested that, although 37cen and 2PI are the major satellite DNA families in the four *Equus* species, other repetitive DNA families exist.

It must be emphasized here that, among horse chromosomes, the only one lacking any signal (both with specific satellites and with the genomic DNA) was ECA 11 and we actually demonstrated, by sequence analysis, that this centromere is totally devoid of satellite tandem repeats [21]. Some of the centromeres lacking any signal in the three other species may actually be completely devoid of satellite repeats, like ECA 11; however, since a molecular characterization of *Equus* centromeres other than ECA 11 is not available, we cannot exclude that short arrays of satellite-type tandem repeats may be present and undetectable by FISH on non-horse *Equus* centromeres. In any case, either the absence or low abundance of tandem repeats at numerous *Equus* centromeres demonstrate that they are characterized by an atypical sequence organization, possibly related to their evolutionary history (see Discussion).

An important observation to be underlined is that, in the present analysis (Figure 2), no 37cen, 2PI or genomic DNA signal was observed on the nine evolutionarily new centromeres that we previously identified in the genus *Equus*, namely the centromeres of ECA 11, EAS 8, EAS 9, EAS 11, EAS 13, EAS 15, EAS 18/EBU 20, EAS 19 [12] and EAS 16/EBU 17 [20].

### Localization of the Centromeric Protein CENP-A and Satellite DNA

**Figure 3:**
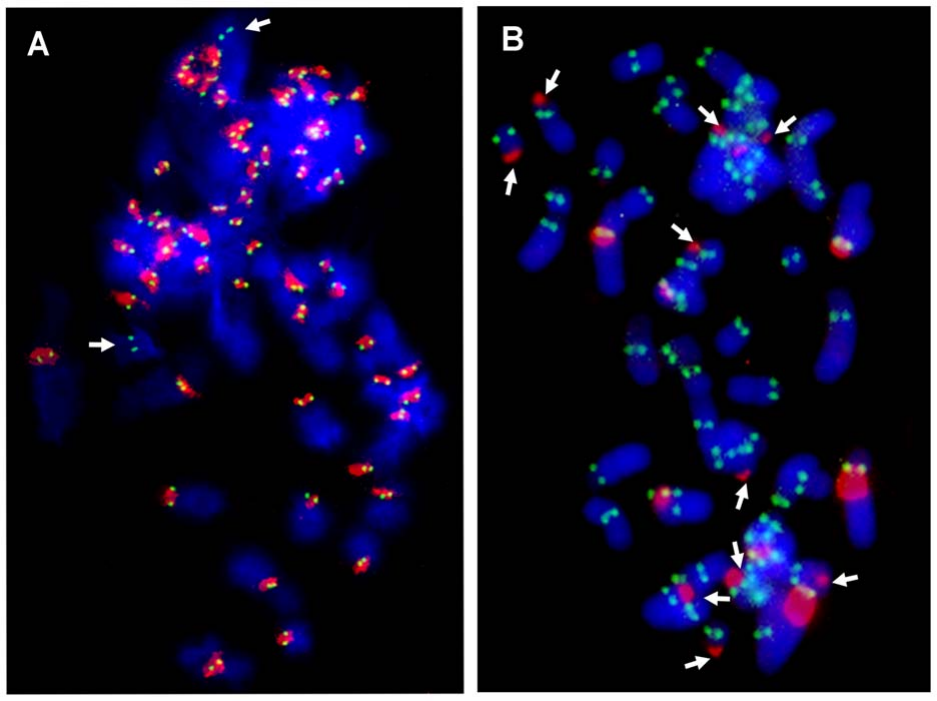
Localization of CENP-A protein and satellite DNA. Immuno-localization of the centromeric protein CENP-A on horse (A) and donkey (B) metaphases was performed by indirect immunofluorescence using a FITC-conjugated secondary antibody (green). Species-specific genomic DNA was used as FISH probe to detect centromeric satellite DNA (red).

In all the horse chromosomes, with the exception of ECA 11, satellite DNA was detected at centromeres (identified as primary constrictions) as in the majority of mammalian species described so far; on the contrary, in the three other *Equus* species, no consistent correlation between the presence of satellite DNA and the primary constriction was observed. In order to confirm that these centromeres are actually sites of centromeric function, we performed immuno-FISH experiments on horse and donkey chromosomes using: 1) an antibody directed against the human protein CENP-A (the H3 histone variant that was previously shown to bind all horse centromeres [21]) for the immuno-identification of centromere function, and 2) horse total genomic DNA, for the localization of satellite DNA (Figure 3). In the horse (Figure 3A) both CENP-A and satellite DNA co-localized on the primary constriction of all chromosome pairs, except ECA 11, which is devoid of satellite DNA and therefore shows only the CENP-A green fluorescent signal. Conversely, in the donkey (Figure 3B), the anti-CENP-A antibody labelled the primary constriction of all the chromosomes, but several centromeres were devoid of satellite DNA, which was instead located at one end of several meta- and submeta-centric chromosomes; on these chromosomes, uncoupling of CENP-A binding and satellite DNA localization was clearly evident.

In conclusion, in the horse, satellite DNA consistently colocalizes with the centromeric protein CENP-A, with the exception of chromosome 11 in which CENP-A but not satellite DNA is present at the centromere; in the donkey, as expected, CENP-A is present at all centromeres (primary constrictions) but satellite signals are often absent at these sites while present at several non centromeric ends.

## Discussion

### Peculiarities of Satellite DNA Localization in the Genus *Equus*

The analysis of the chromosomal distribution of satellite tandem repeats in the four *Equus* species showed that in this genus the organization of such sequences is atypical in two ways: 1) several centromeres seem to be devoid of satellite DNA and 2) satellite repeats are often present at non-centromeric termini (Figure 1 and Figure 2).

The 37cen and 2PI satellites, cloned from horse, represent the two major satellite families in the four *Equus* species. In the horse, either one or both these satellites are present on all chromosomes, except chromosome 11, and only at centromeres. In the other three species, although these satellites are abundant, they are undetectable at several centromeres and tend to be localized at terminal positions. The possibility that other families of satellite DNA may be present at these centromeres was explored by hybridizing the chromosomes with total genomic DNA; this analysis confirmed the presence of highly repetitive tandem arrays in the positions corresponding to those of the 37cen and 2PI probes and demonstrated also the existence of other still non characterized satellites on a few positions (see lower rows in the four panels of Figure 2), in agreement with the early indication obtained by Wichman et al. [28]. Nonetheless, several centromeres still failed to show any satellite hybridization signal. This absence could be due either to the lack of satellite DNA at these sites or to the presence of a number of tandem repeats too low to be detected by FISH.

The total absence of satellite repeats on a centromere has been already proven in one case: ECA 11, at the FISH resolution level, is completely devoid of any satellite DNA signal and the availability of the horse genome sequence assembly allowed us to rule out the presence of any satellite tandem repeat on this primary constriction also at the sequence level [21]. We wondered whether the centromeric function actually resides within the cytogenetically defined primary constriction of ECA 11. An array of this genomic region was hybridized with horse chromatin, cross-linked and immuno-precipitated with an antibody against the kinetochore proteins CENP-A or CENP-C, definitely demonstrating that the centromeric function resides within a DNA sequence totally devoid of satellite DNA [21]. In the same work [21], we also found that ECA 11 showed no accumulation of L1 transposons or KERV-1 elements, which were previously hypothesized to influence ENC formation [29],[30]. Although sequence data are not yet available on other *Equus* centromeres in which satellite DNA is not detectable by FISH, an immuno-FISH analysis with an anti-CENP-A antibody and with satellite DNA showed that, while in the horse satellite DNA and the kinetochore protein co-localize on all chromosomes (with the exception of ECA 11), in the donkey the centromeric function is often uncoupled from satellite DNA (Figure 3). In light of all these observations, it is conceivable that, besides the ECA 11 centromere, other FISH-negative centromeres of donkey and zebras may also be totally devoid of satellite repeats. Although we cannot rule out the presence of short arrays of tandem repeats on FISH negative non-horse *Equus* centromeres, these are nonetheless atypical and are likely to represent evolutionarily “immature” centromeres, that have recently undergone satellite DNA incorporation.

The absence of satellite repeats at some centromeres and their presence at terminal positions are in agreement with our previous observation that several centromere repositioning events occurred during the evolution of the Equidae [12],[20]; in this scenario, these evolutionarily recent events would have generated new centromeres that, at present, are still “immature” and did not yet acquire the sequence complexity typical of the vertebrate centromeres described until now. Conversely, the presence of satellite DNA at terminal positions in meta- and submeta-centric chromosomes, may be interpreted as the trace, left over by centromere repositioning events, of ancient, now inactive, terminal centromeres. In fact, comparative analyses performed using painting probes suggested that the ancestral Perissodactyla karyotype was probably composed of acrocentric chromosomes [19].

**Figure 4:**
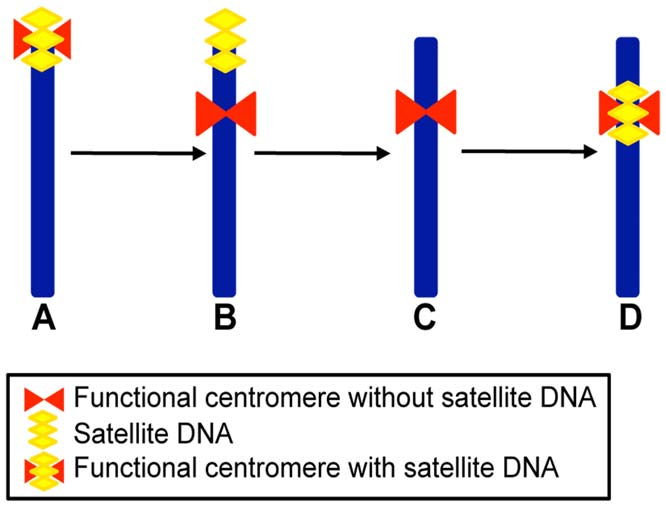
Schematic representation of a four-step mechanism for neocentromere formation during evolution. (A) Acrocentric ancestral chromosome carrying satellite DNA (yellow) at its terminal centromere (red). (B) Submetacentric chromosome derived from centromere repositioning; this chromosome maintained satellite DNA sequences (yellow) at the terminal position, coinciding with the old centromere site, while the neocentromere (red) is devoid of repetitive sequences. (C) Submetacentric chromosome derived from (B) in which the terminal satellite sequences have been lost. (D) Submetacentric chromosome in its full “maturation” stage carrying satellite DNA (yellow) at the neocentromere.

In Figure 4 a schematic representation of the possible steps leading to the formation of meta- or submeta-centric evolutionarily novel centromeres from an acrocentric ancestral chromosome (Figure 4A) is depicted. According to this scheme, and as proposed also by other authors [10],[11],[14], the first step would consist in the shift of the centromeric function to a new position lacking satellite DNA, while the satellite DNA from the old centromere remains in the terminal position (Figure 4B). A subsequent step would be the loss of the terminally located leftover satellite sequences (Figure 4C). The organization of satellite-free immature centromeres may be similar to that of the neocentromeres described in human clinical cases [14]. Finally, the new centromere could reach its maturity by acquiring satellite DNA (Figure 4D) as, for example, in the numerous ENCs described in primates and other species [8]–[13]. Thus, in the case of ECA 11 we may surmise that, while the new centromere did not acquire satellite DNA, the old inactivated centromere lost its satellite repeats, giving rise to a chromosome completely devoid of satellites (as in Figure 4C). The complete or nearly complete loss of satellite sequence from the sites where ancestral centromeres were inactivated could be due to deletion, translocation or recombination events, possibly favoured by the repetitive nature of these sequences. Conversely, it is conceivable that other repositioning events were not followed by the loss of all satellite repeats at the old inactivated centromere, giving rise to chromosomes with satellite repeats at terminal positions only (as in Figure 4B).

### Reconstruction of the Phylogeny of Four *Equus* Chromosomes by Centromere and Satellite DNA Localization

**Figure 5:**
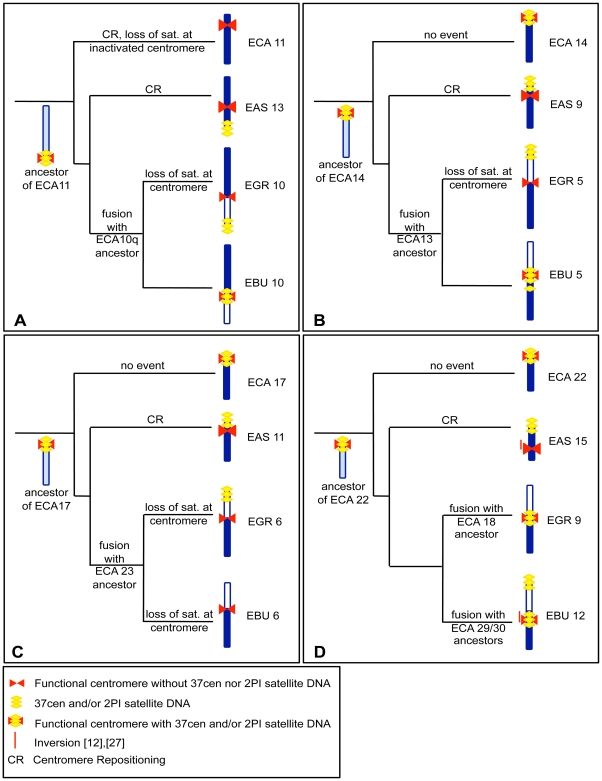
Phylogeny of centromere position and satellite DNA localization in four groups of orthologous chromosomes. The hypothetical events leading to the formation of four groups of orthologous chromosomes from *E. caballus* (ECA), *E. asinus* (EAS), *E. grevyi* (EGR), and *E. burchelli* (EBU) are reported on the branches of the pylogenetic tree of the genus. (A) ECA 11 and its EAS, EGR, and EBU homologs. ; (B) ECA 14 and its EAS, EGR and EBU homologs; (C) ECA 17 and its EAS, EGR, and EBU homologs; (D) ECA 22 and its EAS, EGR, and EBU homologs. Horse chromosomes and their homologous segments in donkey and zebras are colored in dark blue; segments not homologous to the horse chromosomes reported on the left of each row are white colored. Light blue color indicates the configuration of hypothetical ancestral chromosomes. The red bars on the left of EAS 15 and EBU 12 refer to an inversion event. Satellite DNA is drawn in yellow and centromeres in red.

In a previous study [12], we demonstrated that the centromeres of several *Equus* chromosomes derived from repositioning events. This analysis was based on marker order comparisons in *E. caballus*, *E. asinus* and *E. burchelli*. We then used the same markers to extend the analysis to *E. grevyi* (data not shown). In Figure 5 we combined the data on centromere repositioning with the new data on the localization of satellite DNA presented in Figure 1 and Figure 2 of the present work. In these figures the four most informative groups of orthologous chromosomes are represented together with a sketch of the hypothetical ancestral chromosomes and the phylogenetic reconstruction of the events possibly leading to the centromere organization of the chromosomes in the four species.

Figure 5A shows the comparison of ECA 11 with its counterparts in *E. asinus* (EAS 13), *E. grevyi* (EGR 10q) and *E. burchelli* (EBU 10q). As mentioned above, the analysis of marker order on horse chromosome 11 and on the corresponding orthologous chromosomes in *E. asinus* and *E. burchelli* [12], demonstrated that ECA 11 and EAS 13 carry evolutionarily new centromeres. In the present work (see Figure 2A and 2B and Figure 5A) we observed that the two new centromeres lack satellite DNA that is instead localized at the p terminus of EAS 13, at the centromere of EBU 10 and at the p terminus of EGR 10. We hypothesize that the ancestral chromosome from which ECA 11, EAS 13, EGR 10q and EBU 10q derived, was the acrocentric outlined on the left of Figure 5A, containing satellite sequences at its centromere. The centromeric location of this hypothetical ancestral chromosome now corresponds to ECA 11qtel, EAS 13ptel, EGR 10cen and EBU 10cen. In *E. caballus*, the centromere was shifted in its present position, where no satellite DNA is present. The centromere of EAS 13 is also evolutionarily new and lacks any satellite DNA, at the FISH resolution level; the satellite sequences of the now inactive old centromere, have been lost in ECA 11, as in Figure 4C, while they are still present on EAS 13qtel as a relic, as in Figure 4B. Musilova et al. [27], using painting probes, demonstrated that EGR 10p and EBU 10p are orthologous to ECA 10q. It can be supposed that, after the fusion that gave rise to EGR 10 and EBU 10, centromeric satellite DNA was maintained in EBU 10 and lost in EGR 10; alternatively, short arrays of tandem repeats may still be present on the EGR 10 centromere at a level not detectable by FISH. The satellite DNA found on EGR 10ptel might represent the relic of the centromere of an ancestral acrocentric chromosome. Therefore, the absence of satellite DNA is the consequence of an evolutionarily recent repositioning event at the ECA 11 and EAS 13 centromeres, while, at EGR 10 centromere, it is a consequence of the fusion event.

The chromosomes shown in Figure 5B are the orthologs of ECA 14. The ancestral chromosome that presumably gave rise to these chromosomes is represented. Marker order analysis demonstrated that the centromere of EAS 9 was repositioned during evolution [12]. As in the preceding example, the new centromere does not show any satellite FISH signal, while the presence of satellite DNA at EAS 9 short arm terminus might be the fossil evidence of the ancestral centromere position (as in the scheme of Figure 4B). EGR 5 and EBU 5 are probably derived by the fusion of two ancestral acrocentric chromosomes (Figure 5B, right); this hypothesis is confirmed by chromosome painting data [19] which demonstrate that present day ECA 13, EGR 5p and EBU 5p are the orthologs of an acrocentric chromosome in tapirs and rhinoceroses. Presumably, after the fusion, EGR 5 centromere lost satellite DNA while EBU 5 conserved it. The 2PI satellite signal found at the p arm terminus of EGR 5 may be the remnant of an ancestral centromere. The 2PI positive region found in the subcentromeric region of EBU 5 may be the outcome of recombination events involving centromeric repeats.

In Figure 5C, ECA 17 with its donkey and zebra counterparts are shown. The hypothetical ancestral form is reported on the left of Figure 5C. Marker order analysis demonstrated that EAS 11 carries an evolutionarily new centromere [12]. EAS 11 shows no satellite sequences at the centromere while a 2PI positive region is present in the same physical position of the centromere of the ancestral chromosome corresponding to nowadays ECA 14 (as proposed in the scheme of Figure 4B). As in the previous cases, the zebra chromosomes were presumably derived from the fusion of acrocentric chromosomes. The satellite sequences were lost from EGR 6 and EBU 6 centromere. Grevy's zebra chromosome 6 shows satellite DNA signal at the p arm end. Again, this satellite sequence may represent the relic of the centromere of an ancestral acrocentric.

ECA 22 together with its donkey and zebra orthologs are shown in Figure 4D. The arrangement of ECA 22 represents an ancestral organization in mammals [31]. Marker order analysis demonstrated that EAS 15 carries an inversion, encompassed by a red line on the left of the chromosome, and that its centromere is evolutionarily new. The position of the centromere in EBU 12 can be ascribed to an additional zebra-specific centromere repositioning event or to a small inversion (red line on the left of the chromosome) [12]. EAS 15 centromere is devoid of satellite DNA, while the FISH signal present at EAS 15q terminus would represent the relic of the ancestral centromere, as in the scheme of Figure 4B. Both EGR 9 and EBU 12 centromeres were FISH positive. The ancestor of ECA 22, EAS 12, EGR 9q and EBU 12q is sketched on the left in Figure 5D. As hypothesized in the previous examples, the satellite DNA found at EBU 12p end could represent the fossil remains of an ancestral centromere that was inactivated during evolution.

Literature data suggest that the ancestral Perissodactyla karyotype might be very similar to the Rhinocerotidae one, which is characterized by high chromosome numbers (2n = 82−84), most chromosomes being acrocentric [19]. Horse chromosomes 11, 14, 17, and 22 are syntenic to black rhinoceros chromosomes 12, 5, 10 and 25, respectively [19]. These rhinoceros chromosomes are acrocentric; this evidence supports the hypothesis that the satellite DNA found at the non centromeric end of EAS chromosomes carrying ENCs is actually the reminder of the ancestral centromere.

The results presented in this work rise a number of questions concerning the underlying molecular mechanisms. The molecular marks responsible for centromeric function and stability remain elusive, considering that satellite-less centromeres appear to be functional and stable in *Equus* species. While neocentromere formation in human clinical cases is often accompanied by chromosomal rearrangements affecting the normal centromere, it is not clear whether centromere shift during evolution is a consequence of rearrangements of the ancestral centromere leading to loss of function. On the one end, the persistence of satellite DNA at some inactivated centromere sites could simply be a fossil relic or may be maintained by selective pressure. On the other hand, the loss of satellite sequences at some inactivated centromeres, such as the one of ECA 11, could be the consequence of recombination events eliminating functionally irrelevant sequences. Several studies on centromere repositioning in other mammalian orders and in birds [8]–[13] showed that ENCs are apparently less frequent than in the genus *Equus* and that, although evolutionarily novel, they are endowed with satellite sequences. According to the model presented in Figure 4, the *Equus* ENCs are in a still “immature” stage (Figure 4B or 4C), while the previously described ENCs of other orders have acquired satellite DNA reaching “maturity” (stage D in Figure 4). In this scenario, it remains to be established why mature centromeres possess satellite sequences considering that in the genus *Equus* some centromeres can stably function in their absence. Does the mechanics of centromeric function provide a molecular “sink” attracting and conserving repetitive sequences or do such sequences provide some selective advantage to centromere function? All these questions remain open for future investigation that may draw advantage from the study of the rapidly evolving *Equus* centromeres.

### Concluding Remarks

The complex evolution of satellite sequence distribution in the genus *Equus*, observed in the present paper, is in agreement with the instability and exceptional plasticity of the karyotype of these species [16]–[19]. In fact, the centromeric function and the position of satellite DNA turned out to be often uncoupled. Satellite-less centromeres arose from two different evolutionary events: fusions between ancestral acrocentric chromosomes and centromere repositioning. The latter event is unexpectedly frequent in this genus and occurs independently of the acquisition of satellite DNA. This observation supports the hypothesis that large blocks of satellite repeats are not necessarily required for the stability of centromeres. According to this view, satellite repeats may colonize new centromeres at a later stage giving rise to “mature” centromeres according to the pathway schematized in Figure 4. Thus, the rapidly evolving *Equus* species gave us the opportunity to catch snapshots of several ENCs in different stages of “immaturity”.

## Materials and Methods

### Cell Lines and Chromosome Preparation

Fibroblasts were isolated and established from skin biopsies of a male and a female horse and from a male donkey. Grevy's zebra and Burchelli's zebra fibroblasts from female individuals were purchased from Coriell Repositories. Horse, donkey and zebras fibroblasts were cultured in Dulbecco's modified Eagle's medium (CELBIO), supplemented with 20% foetal calf serum (CELBIO), 2 mM glutamine, 2% non essential amino acids, 1x penicillin/streptomycin. Cells were maintained at 37°C in a humidified atmosphere of 5% CO_2_.

For metaphase spread preparation, cell cultures were treated with Colcemid (30 ng/ml, Roche) for 3 h, or mitoses were mechanically collected by direct blowing the medium on the dish surface. Chromosome preparations were performed with the standard air-drying procedure.

### FISH

Whole genomic DNA from horse, donkey, Grevy's and Burchelli's fibroblasts was extracted according to standard procedures [32]. Lambda phage 37cen and 2PI DNA clones, were extracted from 10 ml of bacteria cultures with the Quantum Prep Plasmid miniprep kit (BioRad), according to supplier instructions.

Whole genomic DNA, and 37cen and 2PI satellites, were labelled by nick translation with Cy3-dUTP or Cy5-dUTP (Perkin Elmer) and hybridized to metaphase spreads of primary fibroblasts from the four equid species as described in Nergadze et al. [33]. Briefly, for each slide 250 ng of each satellite, and 25 ng of labelled whole genomic DNA was used. High stringency hybridizations were carried out overnight at 37°C in 50% formamide and post-hybridization washes were performed at 42°C in 2xSSC, 50% formamide; low stringency hybridizations were carried out at 37°C in 25% formamide and post-hybridization washes were performed at 37°C in 2xSSC, 25% formamide. Chromosomes were counterstained with Hoechst 33258. Digital grey-scale images for Cy3, Cy5 and Hoechst fluorescence signals were acquired with a fluorescence microscope (Zeiss Axioplan) equipped with a cooled CCD camera (Photometrics). Pseudocoloring and merging of images were performed using the IpLab software. Chromosomes were identified by computer-generated reverse Hoechst banding according to the published karyotypes.

### Immuno–FISH

Combined immunofluorescence/FISH was performed using a slight modification of the procedure previously describe by Saffery et al. [34]. Fibroblasts were incubated for 2h with 30 ng/ml Colcemid (Roche). The cells were harvested, washed once with phosphate-buffered saline and re-suspended at a concentration of 4×10^4^ cells/ml in 0.075M KCl for 15 minutes at room temperature. 200 µl of cell suspension were cyto-spun (BHG Hermle Z380) onto slides at 1200 rpm for 10 minutes. Slides were incubated in KCM (120 mM KCl, 20 mM NaCl, 10 mM Tris-HCl, 0.5 mM NaEDTA, 0.1% (v/v) Triton X-100) for 15 minute at 37°C and blots air dried. The primary antibody (CENP-A, Upstate) was added and the slides incubated at 37°C for 1 hour followed by three 5 minute washes in KB^-^ (10 mM Tris-HCl, 150 mM NaCl, 1% bovine serum albumin). A FITC conjugated secondary antibody was then added and the slides were incubated for a further hour at 37°C. Two KB^-^ washes were then carried out before fixation in 4% formalin for 15 minutes. Two washes in H_2_0 were carried out and the slides were air dried before further fixation in methanol:acetic acid (3∶1) for 15 minutes. Finally the slides were dried overnight in dark sealed boxes on hygroscopic salts. FISH and immuno-FISH image analysis were performed as described above.
